# Narrow implants (2.75 and 3.25 mm diameter) supporting a fixed splinted prostheses in posterior regions of mandible: one-year results from a prospective cohort study

**DOI:** 10.1186/s40729-017-0102-6

**Published:** 2017-09-08

**Authors:** Tommaso Grandi, Luigi Svezia, Giovanni Grandi

**Affiliations:** 1 Private practice, Via Contrada 323, 41126 Modena, Italy; 2 Department of Obstetrics, Gynecology and Pediatrics, University of Modena and Reggio Emilia, Modena, Italy

**Keywords:** Bone atrophy, Bone resorption, Dental implants, Implant failure, Narrow-diameter implants, Posterior mandible

## Abstract

**Background:**

Can multiple splinted narrow-diameter implants be used as definitive implants in patients with insufficient bone ridge thickness in posterior regions of the mandible? With this aim, we evaluated their outcomes in this set up to 1 year after loading.

**Methods:**

Forty-two patients with a mean age of 61.3 years old (range 49–73) in need of fixed prosthetic implant-supported rehabilitations in the posterior region of the mandible, presenting a thin alveolar crest, were selected. One hundred twenty-four narrow-diameter implants (2.75 and 3.25 mm diameter) were placed and splinted with a bridge. One implant for each missing tooth was requested to be inserted. Outcomes measured were implant survival, complications, and marginal bone level changes up to 1 year after loading.

**Results:**

At the 12-month follow-up, three implants failed. Two 2.75 mm diameter implants and one 3.2 mm diameter implant failed. The implant survival rate was 97.6%. Peri-implant bone resorption was 0.20 mm (CI 95% 0.14: 0.26) after 6 months and 0.47 mm (CI 95% 0.29; 0.65) after 12 months of loading, not different between 2.75 and 3.25 mm diameter groups (*p* = 0.786). Of the 42 cases, three had an episode of peri-implant mucositis (7.1%).

**Conclusions:**

Within the limits of this study, preliminary short-term data (1 year post-loading) suggested that narrow-diameter implants (2.75 to 3.25 mm) can be successfully used as a minimally invasive alternative to horizontal bone augmentation in the posterior mandible. However, larger and longer follow-ups of 5 years or more are needed.

## Background

Historically, implants have been used and documented mainly with diameters between 3.7 and 4.3 mm. Employing these diameters for numerous indications, scientifically substantiated treatment protocols with excellent long-term results have been established [1]. One disadvantage of a standard-diameter implant is the fact that, in clinical use, the available horizontal crestal dimensions of the alveolar ridge are sometimes too small. Although there is some discussion on the amount of bone (buccal and oral) necessary for a successful dental implant, most authors advise at least 1 mm residual bone present adjacent to the implant surface, which consequently requires a horizontal crestal alveolar width of 6 mm for a standard implant. However, the exact threshold for the residual buccal bone thickness has yet not been scientifically clarified and is still under discussion. When inadequate bone width is present for placement of standard-diameter implants, most practitioners have been taught to suggest bone grafting, using either autogenous bone or one of the many available bone substitutes. Bone grafting is a well-documented procedure to restore lost bone volume, but it is associated with increased morbidity and a prolonged treatment time, with the necessary graft-healing period when dentures cannot be worn [2]. While many additive techniques for the reconstruction of missing morphology are employed on a routine basis today, surgical intervention may not always lead to the desired outcome. Physiologically, some patients may be poor candidates for extensive grafting, or they may simply decline such treatment on emotional or financial grounds. Narrow-diameter implants (NDIs) would be beneficial to decrease the rate of augmentations necessary for implant insertion. NDI is an implant with a diameter less than 3.75 mm and is clinically indicated in specific conditions of rehabilitation such as a reduced interradicular bone, thin alveolar crest, or replacing teeth with a small cervical diameter [3]. The availability of residual bone width less than 5 mm is also indicative for the use of NDIs. Several studies have reported the use of narrow-diameter implants in different clinical situations and using different surgical techniques [4–9]. In most cases, satisfactory results have been obtained, achieving medium- and long-term cumulative survival rates equivalent to those obtained in restorations using larger diameter implants (between 94 and 100% survival rates). Until now, the use of NDIs has been restricted to certain defined indications with comparable low occlusal loading like incisors or as retaining elements for overdentures. Posterior regions of the jaws with reduced bone quantity make it challenging to rehabilitate without the use of complex reconstruction techniques.

The aim of this cohort study was to evaluate the outcome of narrow-diameter implants (2.75 and 3.25 mm diameter) used as definitive implants in patients with insufficient bone ridge thickness for placing standard-diameter implants in posterior regions of the mandible. The present study reports the clinical outcome up to 1 year after loading. It is planned to follow up this patients’ cohort to the fifth year of function in order to evaluate the success of the procedure over time. The present article is reported according to the STROBE statement for improving the quality of observational studies (http://www.strobe-statement.org).

## Methods

The present prospective study was conducted at a private practice (Tommaso Grandi, Modena) in Italy between October 2014 and January 2016.

**Fig. 1:**
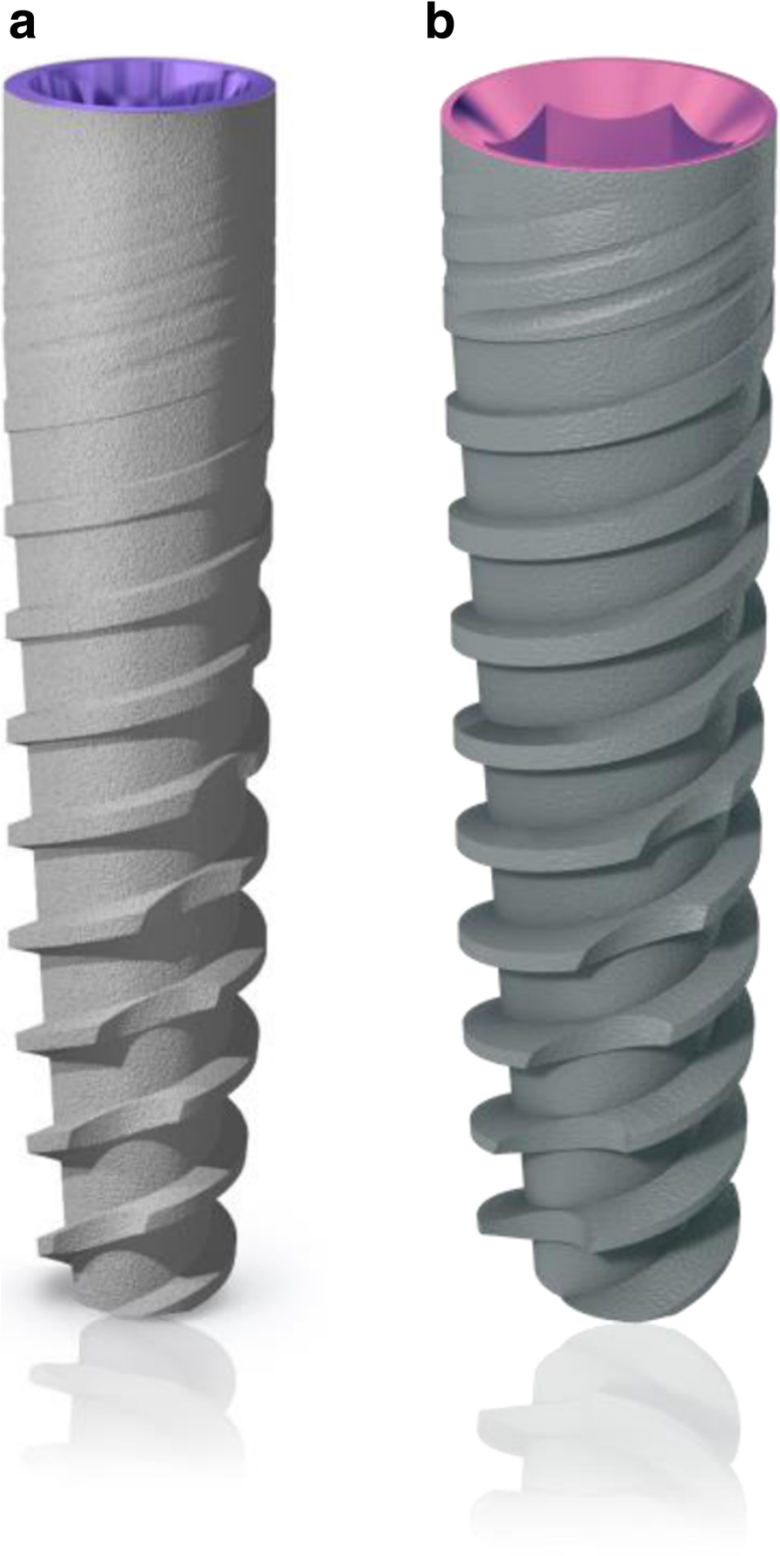
Characteristics of the implants used in the study: **a** external macro-design of JDIcon Ultra S, 2.75 mm diameter implant and **b** external macro-design of JDEvolution S, 3.25 mm diameter implant

Any patient with partial edentulism in posterior regions of mandible (premolar/molar areas), requiring one multiple tooth implant-supported restoration (2-, 3-, or 4-unit bridge), having a residual bone height of at least 8 mm and a thickness of at least 4 mm measured on computerized tomography (CT) scans, and who was 18 or older and able to sign an informed consent form, was eligible for inclusion in this trial. Preoperative periapical X-rays were used for initial screening, followed by computer tomography scans to precisely quantify the amount of bone. Patients were not admitted in the study if any of the following exclusion criteria was present: (1) general contraindications to implant surgery, (2) residual bone thickness greater than 5 mm, (3) subjected to irradiation in the head and neck area, (3) treated or under treatment with intravenous amino-bisphosphonates, (4) poor oral hygiene and motivation, (5) untreated periodontitis, (6) uncontrolled diabetes, (7) pregnant or lactating, (8) substance abusers, and (9) lack of opposite occluding dentition in the area intended for implant placement. The principles outlined in the Declaration of Helsinki on clinical research involving human subjects were adhered to. All patients received thorough explanations and signed a written informed consent before being enrolled in the trial. Forty-two patients were consecutively recruited and treated in a private dental practice by one operator (Tommaso Grandi, who performed all the surgical and prosthetic interventions). All patients underwent at least one session of oral hygiene instructions and professionally delivered debridement when required prior to the intervention. Anti-microbial prophylaxis was obtained with 1 g of amoxicillin and clavulanic acid (Augmentin, Roche S.p.A., Milan, Italy) every 12 h from the day before surgery to the sixth postsurgical day. Patients allergic to penicillin were given clarithromycin 500 mg (Klacid, Abbott srl, Roma, Italy) 1 h before the intervention and 250 mg twice a day for one week. On the day of surgery, patients were treated under local anesthesia. Full-thickness crestal flaps were elevated with a minimal extension to reduce patient discomfort. The implant sites were prepared according to the procedure recommended by the implant manufacturer (JDentalCare, Modena, Italy). Tapered narrow-diameter implants titanium grade 5 (2.75 and 3.25 mm diameter, respectively, JDIcon Ultra S and JDEvolution S, JDentalCare) with internal connection and sandblasted and acid-etched treated surface were used (Fig. 1a, b). No bone flattening was performed. The implants were inserted in the bone without any fenestration/dehiscence. The implant neck was positioned at the coronal marginal crest level. The operator was free to choose implant lengths (8, 10, 11.5, and 13 mm) and diameter (2.75 and 3.25 mm) according to clinical indications. One implant for each missing tooth was requested to be inserted. Healing abutments were attached, and implants were left to a nonsubmerged healing. Interrupted sutures were placed using a synthetic monofilament thread (Vycril, Ethicon, Johnson & Johnson, Somerville, New Jersey) and were removed after 10 days. After 3 months, all the implants underwent the standard prosthetic protocol and were loaded directly with definitive screw-retained or cemented multiple splinted crowns.

Primary outcome measures were as follows:

- Implant failure: evaluated as implant mobility and removal of stable implants dictated by progressive marginal bone loss or infection. The stability of each implant was measured manually by tightening the abutment screw with a wrench delivering a torque of 20 Ncm. Implant stability assessment was performed at delivery of definitive crowns (3 months after implant placement). After insertion of the definitive restorations, prostheses were not removed to assess clinical mobility of individual implants.
- Complications: any biological and prosthetic complication occurred at the implant site during the entire follow-up time were recorded and reported.

Secondary outcome measures were as follows:

- Peri-implant marginal bone level changes: evaluated on intraoral radiographs taken with the paralleling technique at implant placement, 6 months and 1 year after loading. All measurements were taken by an independent assessor (LS). Radiographs were scanned, digitized in JPG format, converted to TIFF format with a 600 dpi resolution, and stored in a personal computer. Peri-implant marginal bone levels were measured using Image J 1.42 software (National Institute of Mental Health, MD, USA). The software was calibrated for every single image using the known implant diameter. Measurements of the mesial and distal crestal bone levels adjacent to each implant were made to the nearest 0.01 mm and averaged at patient level and then group level. The measurements were taken parallel to the implant axis. Reference points for the linear measurements were the most coronal margin of the implant collar and the most coronal point of bone-to-implant contact.

Statistical analysis was performed using the statistical package StatView (version 5.01.98, SAS Institute Inc., Cary, NC, USA). Significance was considered at *p* < 0.05. The paired-samples *t* test was used to evaluate the bone level changes. The patient was the statistical unit of the analysis. A medical doctor (GG) with expertise in dental biostatistics analyzed the data.

## Results

Forty-eight patients were screened for eligibility, but six subjects were not included for the following reasons: five patients (10.4%) were hesitant to receive implant treatment, and one patient (2.1%) was treated with intravenous amino-bisphosphonates. Forty-two patients were then considered eligible and were consecutively enrolled in the study. All patients were treated according to the allocated intervention, no dropout occurred up to 1 year after loading, and the data of all patients were evaluated in the statistical analysis.

Patients were recruited and operated from October 2014 to January 2016.

### Implants and subjects features

The follow-up focused on the time between implant placement and 1 year after loading. One hundred and twenty-four narrow-diameter implants (2.75 and 3.25 mm) inserted in a total of 42 subjects were included. The main baseline patient features are reported in Table 1. Patients were generally healthy, though 19 patients (45.2%) had medication-controlled hypertension and 11 (26.2%) patients had controlled type 2 diabetes. The mean age of the patients at the time of surgery was 61.3 years old (range 49–73). Seating torque values and the dimensions (diameter and length) of the inserted implants are listed in Table 2. Measurements of insertion torque were averaged at patient level and then group level. Average insertion torque was 46.6 Ncm (SD 11.8). Pain and discomfort from the surgical procedure appeared to be within the limits of a flapped implant placement. No incidences of abnormal bleeding or ecchymosis were observed.

**Table 1:** Features of the subjects included in the study

Number of patients	42
Males (%)	18 (42.9%)
Females (%)	24 (57.1%)
Mean age at insertion (range)	62.6 (49–73)
Smokers (less than 10 cigarettes/die)	12 (28.6%)
Diseases in history	
Controlled diabetes type 2	11 (26.2%)
Hypertension	19 (45.2%)
Site of insertion	
Premolar	81 (65.3%)
Molar	43 (34.7%)
Opposite dentition	
Opposing maxillary complete denture	7 (16.7%)
Opposing fixed rehabilitation and natural teeth	26 (61.9%)
Opposing removable prosthesis and natural teeth	9 (21.4%)

**Table 2:** Dimensions (diameter and length) and final seating torque of the inserted implants (*n* = 124)

Length (mm)	8	18 (14.5%)
	10	56 (45.2%)
	11.5	43 (34.7%)
	13	7 (5.6%)
Diameter (mm)	2.75	69 (55.6%)
	3.25	55 (44.4%)
Insertion torque (Ncm)	30	21 (16.9%)
	35	16 (12.9%)
	40	10 (8.1%)
	45	11 (8.9%)
	50	32 (25.8%)
	55	7 (5.6%)
	60	16 (12.9%)
	65	5 (4.1%)
	70	6 (4.8%)

### Implants failures

After 1 year of function, three implants were lost in three patients (one implant per patient) rendering a survival rate of 97.6%. Two 2.75 mm diameter implants and one 3.2 mm diameter implant failed. The failed implants displayed postoperative pain, edema, and signs of infection with pus. They were mobile 3 weeks after placement in smoker women. They were successfully replaced after 4 months.

### Complications

Three patients (7.1%) had an episode of peri-implant mucositis, and they were treated with non-surgical debridement of the affected implants. All permanent bridges remained stable during the 12 months follow-up period.

### Marginal bone level changes

The radiographic data are summarized in Tables 3 and 4. The group lost statistically significant marginal peri-implant bone at 6 months (−0.20; 95% C −0.14: −0.26, *p* < 0.0001) and 1-year post-loading (−0.47; 95% CI −0.29: −0.65, *p* < 0.0001), respectively. The marginal bone level changes were not different between the different implant diameters used, 2.75 and 3.25 mm (*p* = 0.786) (Table 4).

**Table 3:** Comparison of mean bone levels (means ± SD) at different follow-up intervals

Follow-up	Mean bone level (mm) (*n* = 124)	Time	
		0–6 months (95% CI) (*n* = 121)	0–12 months (95% CI) (n = 121)
Baseline	0.01 ± 0.06	−0.20 (−0.14; −0.26)	−0.47 (−0.29; −0.65)
6 months	0.21 ± 0.10	*p* < 0.0001	*p* < 0.0001
12 months	0.48 ± 0.29

**Table 4:** Comparison of mean bone levels (means ± SD) at different follow-up intervals in different implants diameters groups (2.75 and 3.25 mm)

Diameter 2.75 mm				
Follow-up	Mean bone level changes (mm) (*n* = 69)	0–6 months (95% CI) (*n* = 67)	0–12 months (95% CI) (*n* = 67)	p inter-groups
Baseline	0.02 ± 0.07	−0.18 (−0.09; −0.27)	−0.47 (−0.27; −0.67)	p = 0.786
6 months	0.20 ± 0.12	p intra-group		
12 months	0.49 ± 0.30	*p* < 0.0001	*p* < 0.0001	
Diameter 3.25 mm				
Follow-up	Mean bone level changes (mm) (*n* = 55)	0–6 months (95% CI) (*n* = 54)	0–12 months (95% CI) (*n* = 54)	
Baseline	0.00 ± 0.11	−0.22 (−0.10; −0.34)	−0.48 (−0.25; −0.71)	
6 months	0.22 ± 0.14	p intra-group		
12 months	0.48 ± 0.33	*p* = 0.001	*p* < 0.0001	

**Fig. 2:**
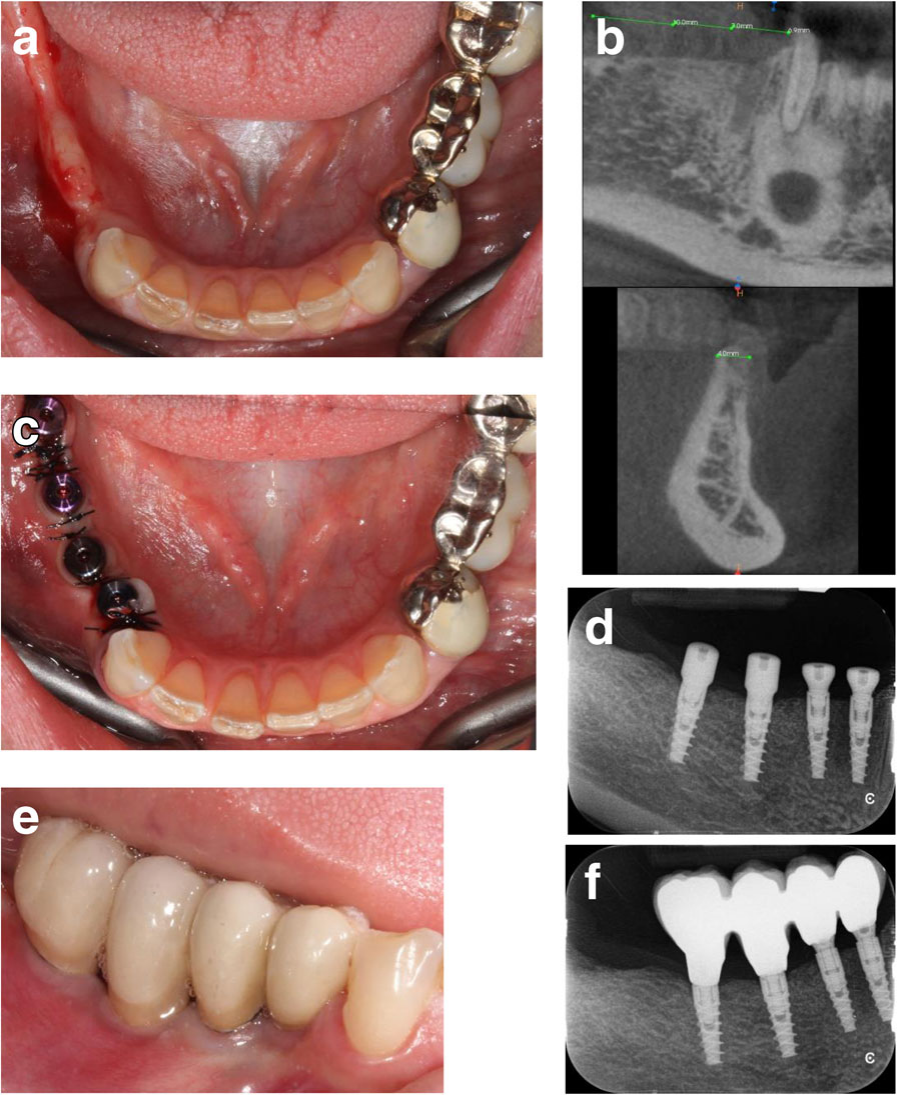
Case 1: Example of one case involved in the study. **a** Preoperative view of a partial edentulism in posterior mandible. **b** Preoperative CT scan. The width of the ridge was 4 mm. **c** Four narrow diameter implants were placed and left to a nonsubmerged healing. **d** Baseline periapical radiograph. **e** Buccal vieew of the final metal ceramic restoration. **f** Periapical radiograph at 1 year after loading

**Fig. 3:**
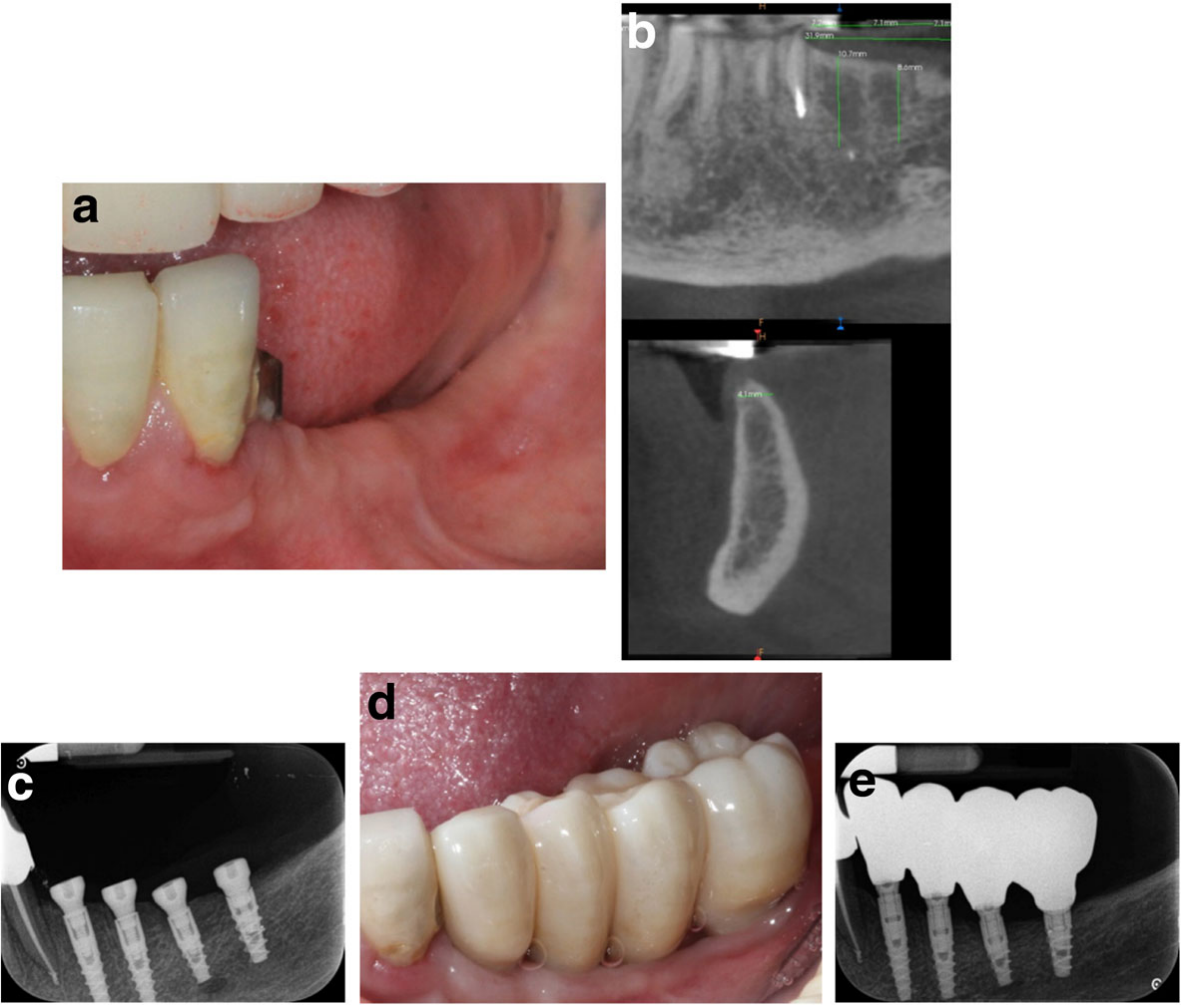
Example of another case involved in the study. **a** Preoperative view –premolars and molars are missing in left mandible. **b** Preoperative CT scan. The width of the ridge was around 4 mm. **c** Baseline periapical radiograph. Four narrow diameter implants were placed to restore the area. **d** Buccal view of the final full-contour zirconia restoration. **e** Periapical radiograph at 1 year after loading

Figures 2 and 3 show the clinical situations before and after treatment in two patients involved in the study.

## Discussion

Dental implants with a reduced diameter are commonly used where bone width is narrow or in cases of restricted mesiodistal anatomy such as laterally maxillary and mandibular incisors. They could also be a viable alternative to bone augmentation especially in challenging situations such as the posterior regions of the mandible. While it has been shown that it is possible to horizontally augment bone in mandible with different procedures, these techniques are associated with significant postoperative morbidity and complications, can be expensive and technique sensitive, and require long treatment periods. Narrow-diameter implants could be simpler, cheaper, and faster alternative to horizontal bone augmentation in the mandible, if they will provide similar success rates. This cohort study was designed to evaluate whether NDIs (2.75 and 3.25 mm diameter) could be used to support partially fixed prostheses in posterior mandibles having insufficient bone ridge thickness for placing standard-diameter implants. At 1-year post loading, implant survival rate was 97.6%, the number of complications was low, and the implants lost an average of 0.47 mm of peri-implant bone. The present data are similar to those observed around other implant systems used in the similar condition. Malo et al. [6] reported a 95.1% survival rate after 11 years of function for narrow-diameter implants (3.3 mm diameter) placed in posterior regions of both jaws. The values for marginal bone resorption recorded in this study at 1, 5, and 10 years (not exceeding 0.2 mm/year of bone loss after the first year) are within the accepted standard success criteria for implants. Regarding the implant failures, the majority occurred in the first 6 months of function, following the pattern for standard-diameter implants. In another retrospective study, Anitua et al. [10] observed a survival rate of 97.3% for 2.5 mm diameter implants used as definitive implants for rehabilitation of missing teeth having a follow-up between 3 and 7 years.

Klein et al., in a recent systematic review, reported that the survival rate of implants with a diameter of < 3 mm was higher than 90% with a follow-up time between 1 and 3 years [3]. In another meta-analysis by Ortega-Oller et al., the majority of the analyzed studies (implants less than 3.3 mm in diameter) have also reported a survival/success rate higher than 90% [11]. However, the results of the meta-analysis have shown higher failure rates for implants with a diameter of < 3.3 mm when compared with implants with a diameter of ≥ 3.3 mm. The authors have related this outcome with the fact that NDIs are usually placed in complicated clinical scenario, and they have a higher possibility of fracture.

On the one hand, due to the small sample size of this study and moreover, the short follow-up (only 1 year after loading), it would be hazardous to conclude that the placement of NDIs to support fixed prostheses in posterior mandible is a predictable treatment modality. In order to draw more reliable conclusions, we need to wait for longer follow-ups, since it may be possible that after several years of function, NDI implants might start to fail due to the reduced available bone-implant contact area or to reduce resistance to fatigue. The placement in the posterior mandible of 2.75 mm diameter implants, as well as 3.25 mm ones, must always be splinted with a bridge, placing one implant for each missing tooth. The placement of a NDI implant in a single molar crown is not recommended. Splinting multiple implants has been reported to minimize the lateral force on the prosthesis, to enhance force distribution, and to reduce the stress on the implants [10]. Thus, splinting of NDI implants would protect the implants from excessive loading and prevent implant/abutment screw fracture. Necessary measures should be taken to minimize off-axis forces like reduction in occlusal table and cusp inclines.

The main limitation of the present study is the small sample size. In addition, a 1-year follow-up is too short to make definitive statements on the predictability of the treatment option tested. Longer follow-up periods and larger sample size are needed, and this trial is currently ongoing.

## Conclusions

Within the limits of this prospective cohort study, narrow-diameter implants (2.75 to 3.25 mm) can be successfully used as a minimally invasive alternative to horizontal bone augmentation in posterior mandible up to 1 year of function. This outcome could be related to the fact that these implants have been all splinted to other implants by a fixed prosthesis. These preliminary results must be confirmed by larger and longer follow-ups of 5 years or more.
